# Effects of novel androgen receptor signaling inhibitors on PSMA PET signal intensity in patients with castrate-resistant prostate cancer: a prospective exploratory serial imaging study

**DOI:** 10.1186/s13550-023-01048-4

**Published:** 2023-10-30

**Authors:** Ida Sonni, Andrei Gafita, Lena M. Unterrainer, Rejah M. Alano, Stephanie Lira, John Shen, Alexandra Drakaki, Tristan Grogan, Matthew B. Rettig, Johannes Czernin, Jeremie Calais

**Affiliations:** 1 Department of Molecular and Medical Pharmacology, University of California, Los Angeles, Los Angeles, CA USA; 2 Department of Radiological Sciences, David Geffen School of Medicine, University of California, Los Angeles, Los Angeles, CA 90095-7370 USA; 3 Department of Experimental and Clinical Medicine, University Magna Graecia, Catanzaro, Italy; 4 Department of Nuclear Medicine, Ludwig Maximilian University of Munich LMU, Munich, Germany; 5 Department of Medical Oncology, University of California, Los Angeles, Los Angeles, CA USA; 6 Department of Medicine Statistics Core, University of California, Los Angeles, Los Angeles, CA USA

**Keywords:** PSMA PET, Hormonal treatment, Androgen receptor, Prostate cancer, PSMA flare

## Abstract

**Background:**

PSMA expression is influenced by hormonal status. We evaluated changes in PSA and whole-body 68Ga-PSMA-11 PET/CT (WB-PSMA PET) after initiation of androgen receptor signaling inhibitors (ARSi).

**Methods:**

Prospectively enrolled patients with metastatic castration-resistant prostate cancer (mCRPC) initiating ARSi underwent serial PSA measurements and WB-PSMA PET at baseline, 1-week, and 3-months post-ARSi. We correlated WB-PSMA PET metrics and PSA kinetics after ARSi to 1-year clinical outcome.

**Results:**

Due to low enrollment rate, the study was closed before reaching the recruitment goal of 30 patients. Nine patients were enrolled. At 1-year, unfavorable outcome was documented in 6/9 (66%) patients. Nine/9 patients completed PSMA PET at 1-week, 5/9 at 3-months. Changes in PSA, PSMA-VOL, SUVmean and SUVmax were − 12%, + 5%, + 3%, and + 10% at 1-week, − 42%, − 16%, − 15% and − 17% at 3-months, respectively.

**Conclusions:**

Our prospective trial involving 9 mCRPC patients initiating ARSi did not show significant modulation of PSMA expression measured on WB-PSMA PET at 1-week. This study was registered on clinicaltrials.gov (NCT04279561).

**Supplementary Information:**

The online version contains supplementary material available at 10.1186/s13550-023-01048-4.

## Background

Androgen receptor (AR) pathway modulation represents a key therapeutic approach for patients with prostate cancer (PCa). AR-targeting treatments are used in case of regional or advanced disease as primary systemic therapy, and as neoadjuvant/concomitant/adjuvant therapy to radiation therapy or surgery in localized or locally advanced PCa [1]. Positron emission tomography (PET) targeting the prostate-specific membrane antigen (PSMA PET) with the two FDA approved radiopharmaceuticals (^68^Ga-PSMA-11 and ^18^F-FCPyL) [2, 3] has become the first-line imaging technique to stage and restage PCa and is recommended by current clinical guidelines (National comprehensive cancer network—NCCN Guidelines—Version 1.2023).

In vitro and in vivo preclinical studies demonstrated that PSMA expression on PCa cells is highly influenced by hormonal status. The folate hydrolase 1 (FOLH1) gene encoding for PSMA synthesis is suppressed by androgens [4–7]. However, clinical studies investigating the effects of AR-targeting treatments on PSMA expression in small cohorts of PCa patients have produced unclear, heterogeneous results [8, 9].

The aim of this study was to evaluate changes on serial whole-body (WB) PSMA PET parameters and serum PSA levels in patients with metastatic castration-resistant prostate cancer (mCRPC) in response to the initiation of a new ARSi.

## Methods

### Study design and patient selection

**Fig. 1:**
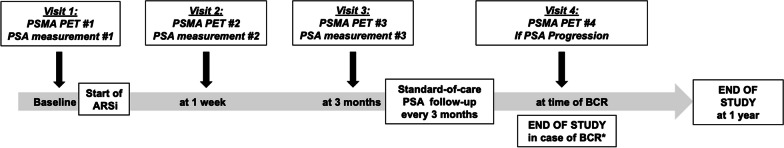
Study design flowchart. *BCR = biochemical recurrence of disease (intended as an increase in serum PSA levels of > 25% and/or an absolute increase of > 2 ng/mL from the nadir documented after initiation of ARSi)

This was a single-center, single-arm, exploratory, prospective study registered on clinicaltrials.gov (NCT04279561). The self-funded study was designed to enroll 30 patients (Fig. 1). The inclusion criteria were: confirmed castrate-resistant prostate cancer (CRPC), known metastatic disease on previous imaging or serum PSA levels ⩾ 1 ng/ml, planned initiation of treatment with AR signaling inhibitors (ARSi, i.e., Enzalutamide, Abiraterone, Darolutamide or Apalutamide). Enrolled patients were required to undergo serial serum PSA measurements and ^68^Ga-PSMA-11 PET/CT (PSMA PET) scans at baseline (before ARSi initiation—visit #1) and at different time points after ARSi initiation: at 1-week (visit #2) and 3-months (visit #3) (details in Additional file 1). After the baseline PSMA PET acquisition (visit#1), patients received the first dose of the prescribed ARSi treatment under the supervision of one investigator. Patients were given directions to continue the daily ARSi treatment, as previously discussed with the treating oncologists. Follow-up with serum PSA measurements was conducted every 3 months after visit #3 (at 6, 9, and 12-months post-ARSi), or until BCR was documented. A 4^th^ and last PSMA PET was required in case of biochemical recurrence (BCR) documented within 1-year from initiation of ARSi. BCR was defined as an increase in serum PSA levels of > 25% and/or an absolute increase of > 2 ng/mL from the nadir documented after initiation of ARSi.

The study was approved by the local ethics committee (UCLA IRB#19-002024).

### Image and outcome analysis

PSMA PET image acquisition parameters and protocol are described in Additional file 1. The image analysis was conducted using the qPSMA software [10] by a board-certified nuclear medicine physician who contoured all PSMA-avid lesions to extract the following quantitative parameters on WB-PSMA PET: PSMA tumor volume (PSMA-VOL), SUVmean and SUVmax. We conducted a patient-level analysis assessing PSA kinetics and changes in WB-PSMA PET parameters at all available time-points. A Wilcoxon signed rank test was used to assess differences over time, and a p-value of 0.05 was considered significant. We defined changes in the quantitative imaging and clinical parameters ≤ 10% as stable and > 10% as clinically significant a priori. A descriptive correlation was obtained for PSA kinetics, PSMA PET parameter changes, and outcome at 1-year post-ARSi.

We classified the clinical PSA outcome at 1-year as follows: favorable, when PSA levels were stable or decreasing from baseline, or unfavorable, when BCR or PCa-related death were documented during follow-up. Serum PSA levels and WB-PSMA PET parameters were considered concordant with clinical outcome in case of an increase with unfavorable outcome or in case of a decrease, or stable values with favorable outcome.

## Results

**Fig. 2:**
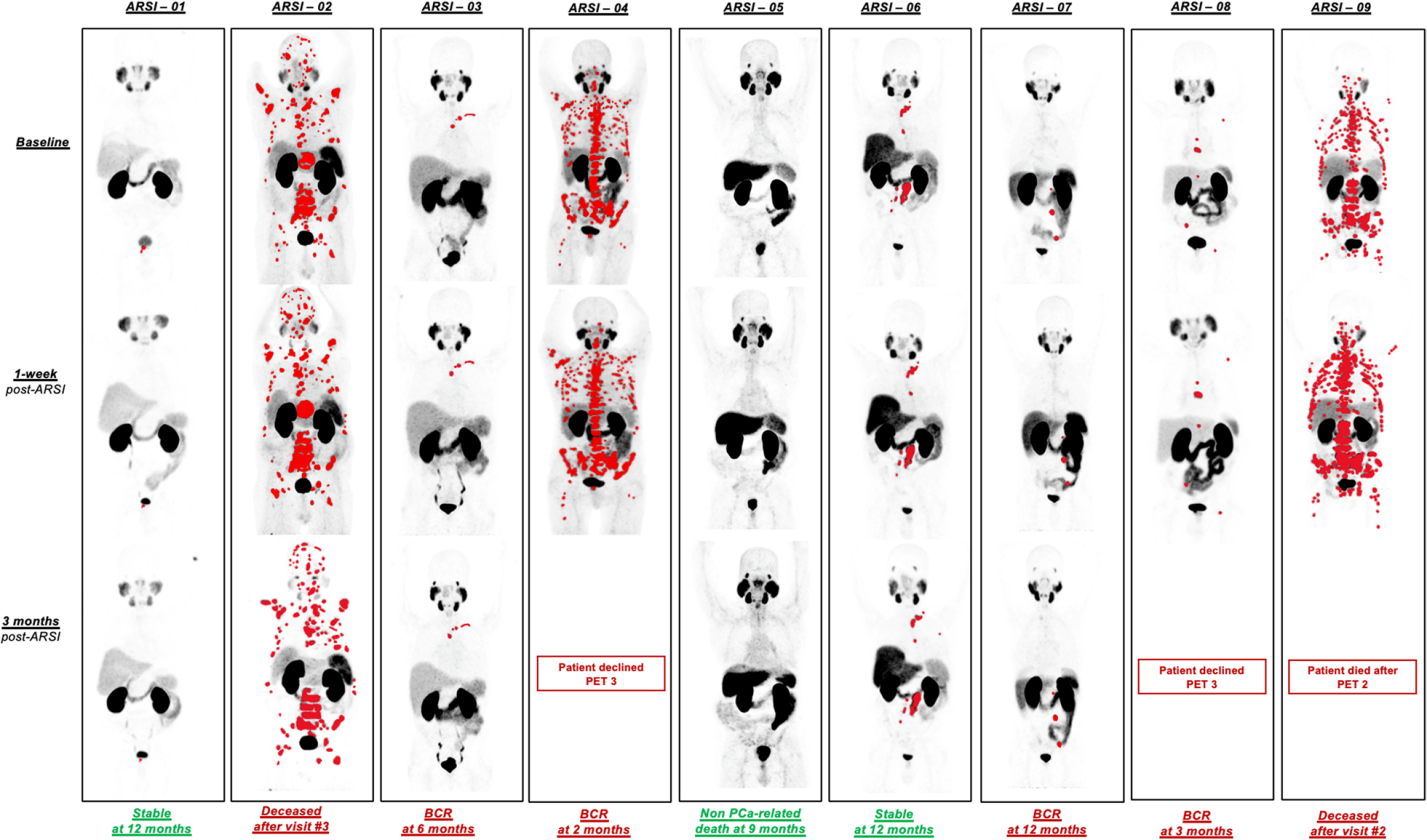
^68^Ga-PSMA-11 PET maximum-intensity projection (MIP) of all patients at different time points with delineated lesions on qPSMA highlighted in red. Patient #02 underwent RT on metastatic bone lesions after PET 2 and died after visit #3. Serum PSA levels and PET metrics should be interpreted in light of the palliative RT. Patient #05 died for non-PCa related causes 9 months after initiation of ARSi

Nine/30 (30%) patients were prospectively enrolled in the study between February 2020 and November 2021. The study was closed on September 15, 2022, due to low enrollment rates and unsatisfactory patient compliance to the protocol. Patients’ characteristics and baseline PSMA PET staging are summarized in Table 1. Five/9 (55%) patients started Enzalutamide, 3/9 (33%) Abiraterone, and 1/9 (11%) Apalutamide. All patients underwent baseline serum PSA measurement and PSMA PET at visit #1 and #2, 6/9 (66%) patients had serum PSA and PSMA PET at visit #3. Two/9 (22%) patients declined to undergo PSMA PET at visit #3. Average changes in PSA and PSMA PET parameters are summarized in Table 2 and PSMA PET analysis in Fig. 2.

**Table 1:** Patient-level changes of PSA and PSMA PET metrics

Patient ID	miTNM at baseline	Type of ARSi	VISIT 1 Baseline				VISIT 2 1-week post ARSi				VISIT 3 3-months post ARSi				Follow-up 6-months	Follow-up 9-months	Follow-up 12-months	1-year outcome
			Serum PSA Baseline	PSMA-VOL	SUVmean	SUVmax	Serum PSA 1-week	PSMA-VOL	SUVmean	SUVmax	Serum PSA 3-months	PSMA-VOL	SUVmean	SUVmax	Serum PSA (6-months)	Serum PSA (9-months)	Serum PSA (12-months)	
ARSI-01	mi T2 N0 M0	Enzalutamide	7.1	13.3	10.9	23.2	3.5 (− 51%)	7.7 (− 42%)	6.9 (− 37%)	12.3 (− 47%)	0.05 (− 99%)	4.4 (− 67%)	4.1 (− 62%)	6.21 (− 73%)	< 0.02	< 0.02	< 0.02	Favorable
ARSI-02	mi T0 N1 M1a M1b	Enzalutamide	72.2	1019	5.4	16.6	60.3 (− 16%)	1309(+ 38%)	5.9 (+ 9%)	22.7 (+ 37%)	34 (− 53%)	1160 (+ 14%)	5.2 (− 4%)	16.95 (+ 2%)	DECEASED			Unfavorable
ARSI-03	mi T0 N0 M1b	Abiraterone	0.2	18.7	4.4	14.6	0.1 (50%)	19.1 (+ 2)	5.2 (+ 18%)	17.5 (+ 20%)	0.05 (− 75%)	17.7 (− 5%)	5 (+ 14%)	13.57 (− 7%)	0.54	0.38	0.64	Unfavorable
ARSI-04	mi T2 N1 M1a M1b	Enzalutamide	11.6	1385	6.8	58.5	9.5 (− 18%)	1536 (+ 11%)	7.3 (+ 7%)	69.9 (+ 19%)	BCR before visit #3—but declined PET #3							Unfavorable
ARSI-05	miT0 N0 M0	Abiraterone	0.33	0	0	0	0.19 (− 42%)	0	0	0	< 0.01	0	0	0	< 0.01	DECEASED due to non-PCa related death		Favorable
ARSI-06	mi T0 N1 M1a	Apalutamide	98.5	136	12	25.7	120 (+ 22%)	140 (+ 3%)	11.7 (− 3%)	25.1 (− 2%)	134 (+ 36%)	127 (− 7%)	10.09 (− 9%)	28.17 (+ 10%)	21.5	7.3	4.2	Favorable
ARSI-07	mi T0 N1 M1a	Abiraterone	2.9	23	11.4	42.7	3.1 (+ 7%)	25(+ 9%)	13.2 (16%)	53.8 (+ 26)	2 (− 31%)	26 (+ 13%)	11 (− 4%)	53.35 (+ 25%)	0.81	n/a	1.15	Unfavorable
ARSI-08	mi T0 N0 M1b	Enzalutamide	17	46	8.2	20.3	12.9 (− 24%)	48 (+ 4%)	8.4 (+ 2%)	23.4 (+ 15%)	25.5 (+ 50%)	BCR at 3 months, but declined PET #3						Unfavorable
ARSI-09	mi T0 N1 M1a M1b	Enzalutamide	67.3	895	7.4	33.1	113 (+ 67%)	1105 (+ 23%)	8.1 (+ 9%)	41.2 (+ 24%)	DECEASED							Unfavorable

**Table 2:** Average and percentage of PSA and PSMA PET metrics in the whole cohort

Clinical and imaging metrics		Visit 1 (*n* = 9)	Visit 2 (*n* = 9)	% change visit 1–2	Outcome at 1-year		p*	Visit 3 (*n* = 5)	% change visit 1–3	Outcome at 1-year		p*
					Unfavorable (*n* = 6)	Favorable (*n* = 3)				Unfavorable (*n* = 2)	Favorable (*n* = 3)	
PSA (ng/mL)	Average (SD)	30.79 (37.74)	35.84 (49.40)	− 12% (0.39)	n/a	n/a	0.82	34.03 (66.66)	− 42% (0.59)	n/a	n/a	0.88
	*n* + 10%	–	2 (#6, #9)	45%	1	1		1 (#6)	36%	–	1	
	*n* stable	–	1 (#7)	7%	1	–		–	–	–	–	
	*n* − 10%	–	6 (#1, #2, #3, #4, #5, #8)	− 34%	4	2		4 (#1, #3, #5, #7)	− 68%	2	2	
PSMA-VOL (mL)	Average (SD)	442.00 (562.69)	523.73 (6676.88)	5% (0.21)	n/a	n/a	0.08	43.78 (56.19)	− 16% (0.35)	n/a	n/a	0.38
	*n* + 10%		3 (#2, #4, #9)	21%	3	–		1 (#7)	13%	1	–	
	*n* stable	–	5 (#3, #5, #6, #7, #8)	5%	3	2		3 (#3, #5, #6)	− 6%	1	2	
	*n* − 10%	–	1 (#1)	− 37%	–	1		1 (#1)	− 67%	–	1	
SUVmean	Average (SD)	8.31 (2.85)	8.34 (2.78)	3% (0.17)	n/a	n/a	0.29	7.75 (3.71)	− 15% (0.33)	n/a	n/a	0.38
	*n* + 10%	–	2 (#3, #7)	17%	2	–		1 (#3)	14%	1	–	
	*n* stable	–	6 (#2, #4, #5, #6, #8, #9)	5%	4	2		3 (#5, #6, #7)	− 6%	1	2	
	*n* − 10%	–	1 (#1)	37%	–	1		1 (#1)	–62%	–	1	
SUVmax	Average (SD)	29.34 (14.91)	33.24 (19.97)	10% (0.06)	n/a	n/a	0.141	25.33 (20.79)	− 17% (0.17)	n/a	n/a	1
	*n* + 10%	–	6 (#2, #3, #4, #7, #8, #9)	24%	6	–		1 (#7)	25%	1	–	
	*n* stable	–	2 (#5, #6)	− 2%	1	1		3 (#5, #3, #6)	1%	1	2	
	*n* − 10%	–	1 (#1)	− 47%	–	1		1 (#1)	− 73%	–	1	

Summary of PSA and PSMA PET quantitative parameters changes at 1-week and 3-months post-initiation of ARSI relative to the 1-year outcome. Patient #5 had no measurable disease on PSMA PET at all time points. Therefore, he was included in the calculations of PSA changes, and changes in PET metrics were considered stable. Patient #2 received palliative RT on two bony metastases after visit #2, and the PSMA PET metrics were not used for the analysis for visit #3. PSA-VOL = whole-body tumor volume

*Wilcoxon signed rank test

### Favorable vs unfavorable outcome at 1-year

Six/9 patients had an unfavorable PSA outcome at 1-year. Four/9 patients experienced BCR, and 2/9, patients #02 and #09, died during the 1-year duration of the study. All patients who experienced BCR declined to undergo PSMA PET at time of BCR. Patient #02 underwent palliative RT on two metastatic bone lesions after visit #2; therefore, the data relative to visit #3 were not included in the analysis.

Three/9 patients had a favorable PSA outcome at 1-year. Patients #1 and #6 had decreasing serum PSA levels at the last time point of the study (1-year post-ARSi initiation). Patient #05 died from a cardio-vascular event at 9-months after ARSi initiation and had unmeasurable serum PSA levels (< 0.01 ng/mL) at the last time point before non-PCa-related death (6-months post-ARSi initiation).

### Correlation of PSA kinetics and changes in PET metrics with 1-year outcome

Of the 6/9 patients with unfavorable PSA outcome at 1-year, all had an increase in at least one PSMA PET parameter, both at 1-week and at 3-months. The 1-week PSA was discordant with 1-year outcome in 4/6 patients, whereas the 3-month PSA was discordant in 3/4 patients.

**Fig. 3:**
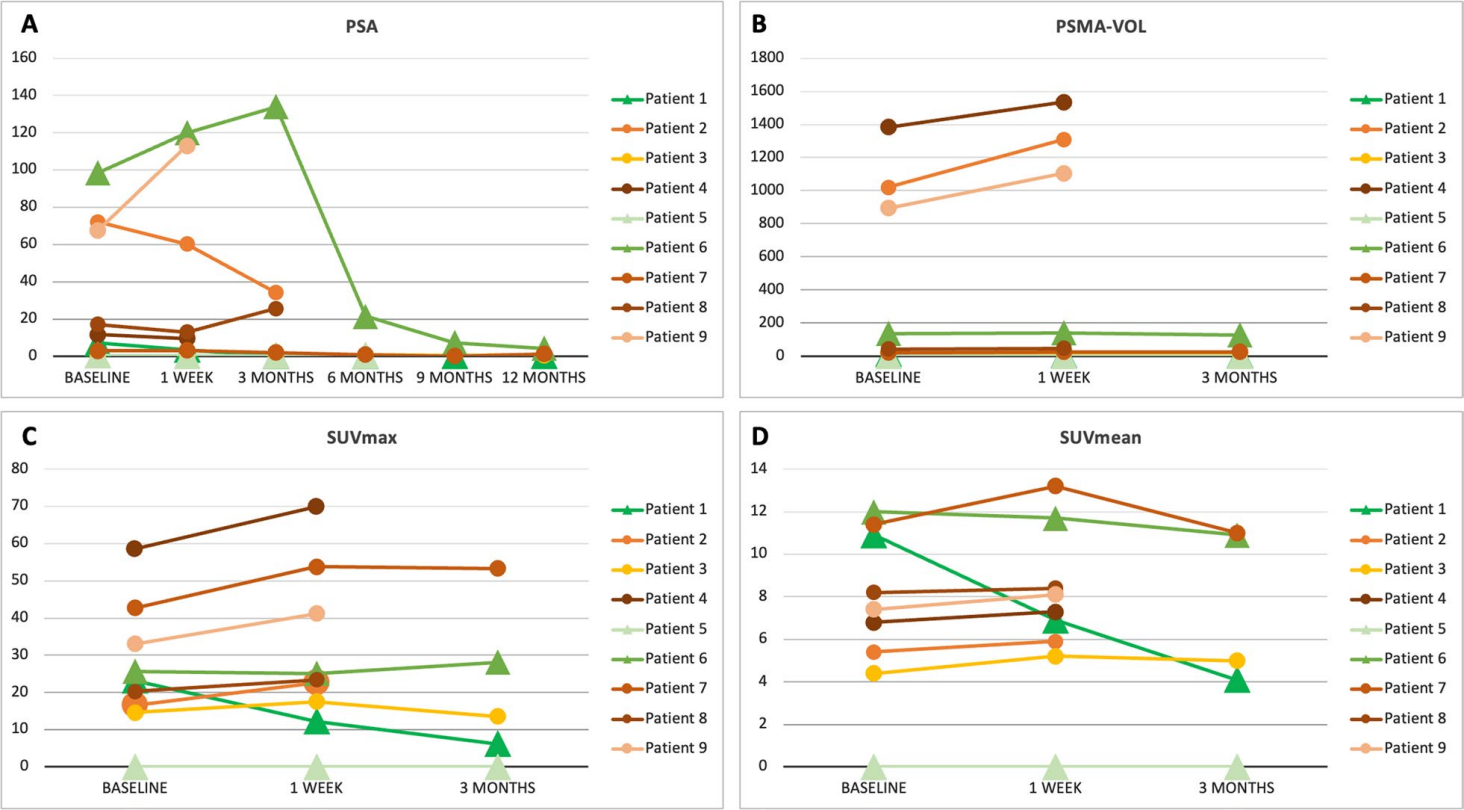
Spaghetti plots showing the serum PSA kinetics (**A**) and the changes in WB PSMA PET quantitative measures (**B**–**D**) for all patients at all time points available. Patients with favorable outcome at 1 year are shown in green colors and with a triangular marker, patients with unfavorable outcome at 1 year are shown in orange and yellow colors, with a circular marker

Of the 3/9 patients with favorable PSA outcome at 1-year, none had increase in any of the PSMA PET metrics at 1-week or 3-months post-ARSi initiation. The PSA changes at 1-week and 3-months were discordant with the 1-year outcome of 1 patient with favorable outcome (Patient #06), who experienced an increase in PSA of 22% and 36% between baseline and week-1, and baseline and 3-months, respectively. The PSA kinetics and the changes in WB PSMA PET metrics are shown in Fig. 3 for all patients and for all available time points.

## Discussion

In this prospective study including nine patients with metastatic castration-resistant prostate cancer (mCRPC) initiating a new drug modulating the androgen receptor signaling (ARSi), we evaluated changes in serum PSA levels and PSMA expression during the initial 3-months after exposure to the new treatment. The goal of our study was to assess whether there is a significant modulation of PSMA expression in response to the androgen receptor pathway inhibition.

At 1-week after ARSi initiation, the whole-body PSMA SUVmean and whole-body tumor volume were stable (changes ≤ 10%) in 6/9 and 5/9 patients, respectively. In our cohort, the average % change of SUVmean, SUVmax, and tumor volume was 3%, 10% and 5%, respectively. This suggests that no significant modulation of PSMA expression induced by ARSi occurs at 1-week in mCRPC.

It was suggested by clinical studies and case reports that a heterogeneous increase in PSMA ligand uptake on PET can be observed in PCa lesions early after initiation of androgen receptor modulating treatments [9, 11, 12]. However, this “PSMA flare” phenomenon has significant inter- and intra-patient variability and was almost exclusively described in castration-sensitive prostate cancer. The mechanism behind this phenomenon is not well understood. A prospective study by Emmet et al. [8] showed that 6 CRPC patients initiating ARSi had a median increase in WB-SUVmax of 45% (IQR: 12.7–66) 9 days after treatment initiation, and a subsequent plateau at later time-points. The authors suggested that the increased PSMA expression may result in a synergistic effect of ARSi and PSMA-targeted radioligand treatments (PSMA RLT). However, in our cohort there was a more heterogeneous response: 1/9 patient had a 47% decrease, 1/9 patient had minimal decrease of 2% (considered stable), and 6/9 had a > 10% increase in SUVmax at 1-week, highlighting patient variability in response to androgen receptor modulation and the need for larger cohorts to demonstrate a significant trend.

While other groups have investigated the effects of ARSi treatments on PSMA expression in patients with prostate cancer and different castration status [8, 9], the novelty of our study was the assessment of PSA kinetics and changes in whole-body PSMA PET quantitative measures in relation to clinical outcome at 1-year after the initiation of the AR modulating treatments. In 6/9 patients, PSA changes at 1-week were discordant with clinical outcome at 1-year, while PSMA PET was concordant. In the remaining 3/9 patients, PSMA PET and PSA kinetics were concordant with each other, and with clinical outcome. Our results show that early PSA monitoring after ARSi initiation is likely not a good prognosticator. While PSMA PET was more often concordant with 1-year outcome than serum PSA levels in our cohort, suggesting the use of serial PET imaging after initiation of ARSi as a prognostic marker appears unrealistic because of cost considerations.

Previous studies have prospectively assessed the effects of ARSi on PSMA expression in mCRPC patients at the whole-body-level [8] and at a lesion-level [9], in 7 and 4 CRPC patients, respectively. The main inherent limitation of doing such analysis is the rigid protocol requiring multiple visits at pre-specified and strict time-points, which interferes with patient compliance. The goal for our study was to prospectively enroll 30 consecutive mCRPC patients initiating a new ARSi treatment, but we were forced to close it early due to recruitment difficulties. Additionally, patients were not encouraged to participate in our study after FDA approval of ^68^Ga-PSMA-11 in December 2020, which allowed easier access to PSMA PET. Despite the recruitment difficulties, our cohort including 9 mCRPC patients was larger than the previously published studies with similar design.

Another intended endpoint of our study was the clinical and imaging assessment of these patients at later time-points, as well as at time of BCR. While all patients underwent PSMA PET at 1-week, 4/9 patients from the initial cohort, all of which had unfavorable outcome, did not undergo PSMA PET at 3-months, leaving the cohort at this time-point skewed toward patients with favorable outcome, and limiting our ability to interpret the 3-months data.

Lastly, we included patients undergoing types of ARSi which may have affected the androgen receptor function, and thus, PSMA expression, differently.

This study contributes to the small body of knowledge on the effects of ARSi on PSMA expression as measured by PSMA PET imaging. We showed that the early impact of these treatments on PSMA modulation is heterogeneous, and likely negligible. Therefore, the manipulations of PSMA levels prior to RLT may not be warranted early after ARSi.

## Conclusion

In this exploratory study that prospectively enrolled 9 patients with metastatic castration-resistant prostate cancer initiating a new ARSi, we found a heterogeneous response to the AR pathway modulation and in most cases discordance between PSA kinetics and whole-body PSMA PET measurements. The modulation induced by ARSI to the PSMA expression of the whole tumor burden of mCRPC patients was not observed at 1-week. A larger cohort is needed to confirm the trend highlighted in our study.

### Supplementary Information

**Additional file 1.** Patient demographics. n/a = not applicable.

## Data Availability

The datasets generated during and/or analyzed during the current study are available from the corresponding author on reasonable request.

## Abbreviations

AR: Androgen receptor
ARSi: AR signaling inhibitors
BCR: Biochemical recurrence
CRPC: Castrate-resistant prostate cancer
FDA: Food and Drug Administration
FOLH1: Folate hydrolase 1
mCRPC: Metastatic castration-resistant prostate cancer
NCCN: National comprehensive cancer network
PCa: Prostate cancer
PET: Positron Emission Tomography
PSA: Prostate-specific antigen
PSMA: Prostate-Specific Membrane Antigen
PSMA PET: Positron Emission Tomography/Computer Tomography with ^68^Ga-PSMA-11
PSMA-VOL: Tumor volume on PSMA PET
SUVmax: Maximum standardized uptake value
SUVmean: Mean standardized uptake value
WB: Whole-body
