# Supplementary material for: Effects of novel androgen receptor signaling inhibitors on PSMA PET signal intensity in patients with castrate-resistant prostate cancer: a prospective exploratory serial imaging study
Source: EJNMMI Res. 2023 Oct 30;13:95. doi: 10.1186/s13550-023-01048-4 (PMC10616012; doi:10.1186/s13550-023-01048-4)
Supplement: Supplementary file 1 — Additional file 1. Patient demographics. n/a = not applicable. [file 13550_2023_1048_MOESM1_ESM.docx]

**Effects of novel androgen receptor signaling inhibitors on PSMA PET signal intensity in patients with castrate-resistant prostate cancer: a prospective exploratory serial imaging study**

Authors:  Ida Sonni^1,2,3,^ Andrei Gafita^1^, Lena M. Unterrainer^1,4^, Rejah M. Alano^1^, Stephanie Lira^1^, John Shen^5^, Alexandra Drakaki^5^, Tristan Grogan^6^, Matthew B. Rettig^5^, Johannes Czernin^1^, and Jeremie Calais^1^

1. Department of Molecular and Medical Pharmacology, University of California, Los Angeles, Los Angeles, CA, USA
2. Department of Radiological Sciences, University of California, Los Angeles, Los Angeles, CA, USA
3. Department of Experimental and Clinical Medicine, University Magna Graecia, Catanzaro, ITALY
4. Department of Nuclear Medicine, Ludwig Maximilian University of Munich LMU, Munich, GERMANY
5. Department of Medical Oncology, University of California, Los Angeles, Los Angeles, CA, USA
6. Department of Medicine Statistics Core, University of California, Los Angeles, Los Angeles, CA, USA

**Word count: 2657**

**Running title: PSMA modulation by ARSi**

**Correspondence to**: Ida Sonni, MD; Department of Radiological Sciences, David Geffen School of Medicine; University of California, Los Angeles, Los Angeles, CA, USA 90095-7370

**E-mail**: [isonni@mednet.ucla.edu](mailto:isonni@mednet.ucla.edu); ORCID ID: 0000-0002-8802-7704

**Keywords**: PSMA PET, hormonal treatment, androgen receptor, prostate cancer, flare phenomenon

**SUPPLEMENTAL MATERIAL**

**METHODS**

**Study workflow**

After referral by their treating medical oncologists and confirmation of fulfillment of the inclusion/exclusion criteria, enrolled patients came to the UCLA nuclear medicine clinic for their baseline assessment (Visit #1). Patients underwent a blood withdrawal for serum PSA measurement, and a PSMA PET scan. At the end of the PET acquisition, patients took the first dose the prescribed ARSI treatment under the supervision of one of the investigators and were instructed to return for visit #2 (1 week after). The patients were given directions to continue with the ARSI treatment on a daily basis, as previously discussed with the treating oncologists. During Visit #2 (1 week after ARSI initiation) and visit #3 (3 months after ARSI initiation), patients underwent a blood withdrawal for PSA measurement, and a PSMA PET scan. Serum PSA measurements were continued every three months after visit #3, until BCR was documented.

**PSMA PET acquisition and protocol**

The PSMA-targeting ligand used for the PSMA PET was 68Ga-PSMA-11 (Glu-NH-CO-NH-Lys-(Ahx)-[68Ga(HBED-CC)]) (*1*). Images were acquired using a 64-detector PET/CT scanner (2007 Biograph 64 Truepoint or 2010 Biograph mCT 64; Siemens, Munich, Germany). A non-diagnostic, low dose CT scan was obtained was obtained for all PSMA PET/CT scans to reduce exposure to radiation for repetitive scans.

A whole-body scan was acquired from pelvis to vertex prior to a dedicated post-void pelvic scan. The latter was not used for the analysis. All PET images were reconstructed with corrections for attenuation, dead-time, random events, and scatter, using iterative ordered-subsets expectation. The time per bed position was based on patient weight (*2*).

| Total number of patients | 9 |
| --- | --- |
| Median Age, yr (IQR) | 74 (69 – 79.5) |
| Median PSA, ng/mL (IQR) | 11.1 (7.5 – 21.5) |
| Primary therapy, n (%)   - - Surgery   - Radiation Therapy   - ADT | - - 3 (33)   - 4 (44)   - 2 (22) |
| First line ADT, n (%)   - - Lupron   - Lupron + Casodex   - Lupron + Abiraterone   - Degarelix + Denosumab   - Goserelin   - unknown | - - 2 (22)   - 2 (22)   - 2 (22)   - 1 (11)   - 1 (11)   - 1 (11) |
| Pre-surgical Gleason Grade, n (%)   - - 3+3=6   - 3+4=7   - 4+3=7   - 4+4=8   - 5+4=9   - unknown | - - 2 (22)   - 1 (11)   - 1 (11)   - 1 (11)   - 2 (22)   - 2 (22) |
| Salvage therapy type, n (%)   - - Salvage Radiation therapy   - n/a | - - 5 (55)   - 4 (44) |

**Table 1:** Patient demographics. n/a = not applicable

**REFERENCES**

**1.** Eder M, Schäfer M, Bauder-Wüst U, et al. 68Ga-complex lipophilicity and the targeting property of a urea-based PSMA inhibitor for PET imaging. *Bioconjug Chem.* 2012;23:688-697.

**2.** Halpern BS, Dahlbom M, Quon A, et al. Impact of patient weight and emission scan duration on PET/CT image quality and lesion detectability. *J Nucl Med.* 2004;45:797-801.
